# Animal Models of Leptospirosis: Of Mice and Hamsters

**DOI:** 10.3389/fimmu.2017.00058

**Published:** 2017-02-21

**Authors:** Maria Gomes-Solecki, Ignacio Santecchia, Catherine Werts

**Affiliations:** ^1^Department of Microbiology, Immunology and Biochemistry, The University of Tennessee Health Science Center, Memphis, TN, USA; ^2^Institut Pasteur, Unité Biologie et Génétique de la Paroi Bactérienne, Paris, France; ^3^INSERM, équipe Avenir, Paris, France

**Keywords:** *Leptospira interrogans*, leptospirosis, animal models, hamsters, mouse models, virulence factors, TLR4, TLR2

## Abstract

Pathogenic *Leptospira* sp. are spirochetal bacteria responsible for leptospirosis, an emerging worldwide zoonosis. These spirochetes are very successful pathogens that infect a wide range of hosts such as fish, reptiles, birds, marsupials, and mammals. Transmission occurs when chronically infected animals excrete live bacteria in their urine, contaminating the environment. *Leptospira* sp. enter their hosts through damaged skin and mucosa. Chronically infected rats and mice are asymptomatic and are considered as important reservoirs of the disease. Infected humans may develop either a flu-like, usually mild illness with or without chronic asymptotic renal colonization, or a severe acute disease with kidney, liver, and heart failure, potentially leading to death. Leptospirosis is an economic burden on society due to health-care costs related to elevated morbidity of humans and loss of animals of agricultural interest. There are no effective vaccines against leptospirosis. *Leptospira* sp. are difficult to genetically manipulate which delays the pace of research progress. In this review, we discuss in an historical perspective how animal models have contributed to further our knowledge of leptospirosis. Hamsters, guinea pigs, and gerbils have been instrumental to study the pathophysiology of acute lethal leptospirosis and the *Leptospira* sp. genes involved in virulence. Chronic renal colonization has been mostly studied using experimentally infected rats. A special emphasis will be placed on mouse models, long thought to be irrelevant since they survive lethal infection. However, mice have recently been shown to be good models of sublethal infection leading to chronic colonization. Furthermore, congenic and transgenic mice have proven essential to study how innate immune cells interact with the pathogen and to understand the role of the toll-like receptor 4, which is important to control *Leptospira* sp. load and disease. The use of inbred and transgenic mouse models opens up the field to the comprehensive study of immune responses to *Leptospira* sp. infection and subsequent pathophysiology of inflammation. It also allows for testing of drugs and vaccines in a biological system that can avail of a wealth of molecular tools that enable understanding of the mechanisms of action of protective vaccines.

## Introduction

The most important factor in the development of animal models of leptospirosis is that experimental infection closely recapitulates natural disease in humans. Only then, these tools can be used in fundamental research of *Leptospira* sp. pathogenesis and disease, host–pathogen interactions leading to eradication or persistence of *Leptospira* sp., characterization of pathogen associated virulence factors, immune responses to infection and subsequent pathophysiology of inflammation. Under the realm of applied research, these models can be used to test vaccines to prevent infection or disease progression and to test therapeutics for cure or to mitigate signs and symptoms of the illness. Given the limited availability of properly validated biological samples from human leptospirosis patients, animal models also provide a source of material (especially urine) than can be used to develop proof-of-principle versions of new diagnostic assays.

We start the review by defining the enzootic cycle of pathogenic *Leptospira* sp. and the clinical presentation of the disease in human patients to frame how animal models that address distinct components of the cycle contribute to the understanding of how reservoir hosts contaminate the environment and enable transmission of pathogenic *Leptospira* sp. to humans and how we can use these animals to better understand disease pathogenesis. We describe the animal models used to study the forms of lethal, sublethal and chronic leptospirosis with an emphasis on mouse models.

The mouse is a versatile animal model to study *Leptospira* sp. infection because we can avail of a vast number of reagents and genetic backgrounds tailored to providing answers to specific questions.

### The Enzootic Cycle of Pathogenic *Leptospira* sp.

Leptospirosis is an emerging zoonotic disease with a worldwide distribution caused by infection with any of the several pathogenic serovars of *Leptospira* sp. The disease affects virtually all vertebrates and has a broad range of clinical signs and symptoms, from mild, subclinical infection to multiple-organ failure and death. *Leptospira* sp. penetrate abraded skin or mucous membranes, enter the bloodstream, and disseminate throughout the body. The pathogens are easily maintained in sylvatic and domestic environments mostly by transmission through rodent species. In these reservoirs, infection produces chronic, asymptomatic carriage. Some pathogenic *Leptospira* sp. such as Canicola and Hardjobovis are maintained in non-rodent mammal reservoirs. *Leptospira* sp. can then infect livestock and domestic and wild animals and cause a range of disease manifestations and carrier states. Maintenance of *Leptospira* sp. in these populations is due to their continued exposure to animal reservoirs or to transmission within animal herds. Accidental hosts like humans can be infected by direct contact with reservoir animals or by exposure to environmental surface water or soil that is contaminated with their urine (1).

### Clinical Presentation of the Disease in Human Populations

Human leptospirosis ranges in severity from a self-limited febrile illness to a fulminant life-threatening illness, also called Weil’s disease. When illness occurs, a broad array of organ systems may be involved, reflecting the systemic nature of the infection. As a result, the signs and symptoms of leptospirosis are frequently mistaken for other causes of acute febrile syndrome (2). A recent systematic review of published cases estimated that leptospirosis causes ~1 million cases a year resulting in ~6% death rate (3).

Most leptospirosis cases are mild and resolve spontaneously (>90%). It typically presents as a biphasic disease, with an initial acute illness lasting about 1 week characterized by fever, myalgia, and headache that may be confounded with other entities such as influenza and dengue fever. In this phase, *Leptospira* sp. are found in blood or in the cerebrospinal fluid. The second phase is characterized by the presence of *Leptospira* sp. in urine and the immune response to *Leptospira* sp. is detectable by traditional serological methods. A low percentage of patients (<10%) progress to multisystem organ failure and have widespread hematogenous dissemination of pathogens resulting in non-oliguric (high-output) renal dysfunction or oliguric renal failure (2, 4). Hemorrhagic complications are common and are associated with coagulation abnormalities. Severe pulmonary hemorrhage syndrome due to extensive alveolar bleeding has a fatality rate of >50% (2).

If we consider the clinical outcomes in human populations described above, we can group leptospirosis as sublethal and lethal infections. Given the most recent numbers (3) on mortality rates in humans afflicted with leptospirosis, it is reasonable to expect that >90% of people go on to develop sublethal cases of the disease. In lethal forms of the disease, either the kidney or the lung are the affected organs. In addition, patients may also present altered mental status due to neuroleptospirosis (5, 6), and liver and other organs may also be involved. Although leptospirosis is primarily a zoonosis, with humans considered as accidental hosts, it is worth noting that transient *Leptospira* sp. shedding does occur during human infection and human-to-human infection, although extremely rare, has been reported (2, 7, 8). Moreover, chronic asymptomatic leptospirosis has recently been shown in a Peruvian population (9), suggesting that the renal colonization is not a peculiarity of some animal carrier but rather it may follow severe acute leptospirosis. Thus, there is a wealth of information to be learned from sublethal and/or chronic models of *Leptospira* sp. infection.

## Physiopathology of Animal Leptospirosis

In this part, we discuss in an historical perspective the use of animal models of leptospirosis to better understand the physiopathology of the disease, with an emphasis on the mouse model that has been largely overlooked.

### Historic Perspective on the Use of Animal Models of Leptospirosis

The first animal model of acute *Leptospira* sp. infection and disease was reported by Inada and colleagues in 1916. They injected blood from a patient with Weil’s disease into monkey, rabbit, rat, and guinea pig and observed that 7 days post-infection only the guinea pig developed signs and symptoms consistent with Weil’s disease. They followed up with microscopic examination of the liver and detected a bacterium morphologically identical to the spirochete they observed in specimens of blood, intestinal walls, and adrenal glands obtained from patients who succumbed to the disease. They concluded that this spirochete was the pathogenic cause of Weil’s disease and named it *Spirochaeta icterohaemorrhagiae* (10).

In the mid-twentieth century, it was reported that golden Syrian hamsters were particularly susceptible to *Leptospira* sp. infection (11). Guinea pigs, golden hamsters, and dogs were then used for laboratory studies of pathogenic *Leptospira* sp. infection (12–15). Over the same period, it was also shown that young white mice were extremely susceptible to infection with *Leptospira interrogans* serovar Icterohaemorrhagiae (16) and that different strains of mice varied greatly in susceptibility to this organism (17). Others reported that mice were susceptible to some of the *Leptospira* sp. serotypes isolated in Northern Australia and that survivors became permanent renal carriers (18). In 1963, a lack of availability of golden Syrian hamsters in Australia led Spradbrow to establish a model of *Leptospira* sp. infection in mice (15–20 g, ~6 weeks old), which produced acute disease with over 50% of mortality and chronic persistent renal infections in the surviving animals. To address the need for complete cure of carriers of chronic renal infections, Spradbrow used this model to study the effectiveness of antibiotic treatments in clearance of *Leptospira* sp. from urine and kidney and found that streptomycin was the only antibiotic of which a single administration regularly cured chronic renal infections (19). Interestingly, as recently as 2001, streptomycin was found to be the most effective anti-*Leptospira* sp. antibiotic in patients diagnosed with Weil’s disease (20).

Although guinea pigs and golden hamsters were the animals most commonly used in laboratory studies of *Leptospira* sp. infections in the mid-twentieth century, it was also recognized that both species are less convenient to handle than mice, which are the ideal laboratory animal. Fast forward 50 years and the same questions are debated today worldwide. For example, in the USA, the Animal Welfare Act (AWA) (7 U.S.C. § 2131) is the federal law that regulates the care and use of animals in research. The AWA provides protections for certain species (such as hamsters, guinea pigs, dogs, and non-human primates) while excluding others such as mice (*Mus* sp.) and rats (*Rattus* sp.) bred for research. The fact that guinea pigs and hamsters are covered under AWA protected species leads to onerous regulation (housing, medical records, transportation) and to United States Department of Agriculture official yearly inspections, which in turn results in a considerably lower use of these animals in US laboratories (5–10% use of all protected species). Cumbersome regulations provide the global research community with an opportunity to develop additional mouse or rat models of research for leptospirosis.

### Lethal Leptospirosis in Hamsters and Guinea Pigs

Throughout the last quarter of the twentieth century, hamsters have been used as the primary model for acute leptospirosis. The route of infection is via intraperitoneal injection, usually with *Leptospira* sp. resuspended in EMJH medium. Young hamsters (4 weeks old) infected with a broad range of pathogenic serovars of *Leptospira* sp. develop fulminant, disseminated infection, which reproduces the severe form of human leptospirosis with the presence of *Leptospira* sp. in the tissues, destruction of hepatocyte junctions that leads to jaundice, leukocytosis, hemorrhage, endothelial alteration, thrombotic glomerulopathy, and interstitial nephritis (21–23). Hamsters are desert animals traditionally not exposed to the humid conditions that mediate transmission of *Leptospira* sp. Possibly for that reason, hamsters are exquisitely sensitive to leptospirosis since one single organism is able to cause disease (23). The severe leptospirosis induced by pathogenic *Leptospira* sp. in hamsters is associated with enhanced expression of pro-inflammatory cytokines and mediators by peripheral blood cells, such as IL-10, IL-1α, TNF-α, and the cyclo-oxygenase 2 (24). Upon infection with *L. interrogans* serovar Icterohaemorrhagiae strain Verdun, mRNA levels of these immunomodulators have been found to be higher in hamsters that will not survive the infection compared to survivors (25). Also, the intensity of the pro-inflammatory response varies according to the strain used, as do the bacterial loads in organs. Hence, infection with the highly virulent (V) serovar Manilae generates higher levels of cytokine transcripts in lungs and liver compared to the infection with *L. interrogans* Hebdomadis (26). These data suggest that the uncontrolled cytokine production found in hamsters upon infection with pathogenic *Leptospira* sp. may mimic the adverse cytokine storm described in sepsis.

Common applications of the hamster model of leptospirosis include determination of strain infectivity, routes of infection (27), restoring virulence to culture-attenuated strains, assessing usefulness of potential vaccines or diagnostic antigens, and examining pathology of kidney (23).

Severe pulmonary leptospirosis has been studied using the guinea pig model (28) as it replicates the pulmonary hemorrhage and respiratory failure seen in humans. Studies of this model revealed thrombocytopenia, extensive hemorrhage of the lungs, absence of intravascular coagulation, and extensive deposition of immunoglobulin and complement along the alveolar basement membrane, which suggested that an immune process may be involved in the etiology of fatal pulmonary hemorrhage in leptospirosis (28, 29). The same pathology was replicated in dogs, which could also be used as a natural disease model for human leptospirosis (30).

### Mouse Models of Leptospirosis

Rats and some strains of mice (31) are generally unsuitable hosts for acute lethal leptospirosis because they develop severe signs of disease only within a short window of time after birth, before 3–4 weeks, but afterward no longer succumb to infection (32). This suggests that a mature immune system achieved by 5 weeks of age in mice (33) is required to control leptospirosis to ensure survival. However, a number of inbred wild-type (WT), immunosuppressed, or transgenic mice have been used as models of lethal, sublethal and chronic leptospirosis. A summary of mice as animal models to study leptospirosis is provided below and in Table 1.

**Table 1:** **Mice as animal models to study disease caused by pathogenic *Leptospira* sp**.

Mouse strain	Genotype	Model of disease	Pathogenic *Leptospira* sp. serovar and strain	Biological variables			Tissue dissemination (technique, dpi)	Major findings	Reference
				Sex	Age (w.o.)	Infection route/dose			
A/J	*ahl4* mutation	Asymptomatic	Icterohaemorrhagiae strain Cop	F	4–5	IP/10^3^, 10^6^	Kidn (IF, dpi 28)	High kidney colonization	Santos et al. (34)
CBA	*pde6b* mutation	Asymptomatic	Icterohaemorrhagiae strain Cop	F	6–7	IP/10^3^, 10^6^	Kidn (IF, dpi 28)	Inflammatory lesions and interstitial nephritis	Santos et al. (34)
BALB/c	Wild type (WT)	Asymptomatic	Copenhageni strain Cop	ND	ND	IP/10^6^, 10^7^	Kidn (HP, dpi 28)	No signs of disease	Bandeira et al. (35)
	CB17 SCID	Acute					Kidn, Liv, lun (HP, dpi 28)	Pulmonary hemorrhage	
C57BL/6	WT	Asymptomatic	Icterohaemorrhagiae strain Cop	F	4–5	IP/10^3^, 10^6^	Kidn (IF, dpi 28)	Inflammatory lesions and interstitial nephritis	Santos et al. (34)
	WT	Asymptomatic	Copenhageni strain Cop	ND	ND	IP/10^3^, 10^6^ IP/10^6^, 10^7^	Lun, Kidn (HP, dpi 28)	Interstitial nephritis	Bandeira et al. (35)
	iNOSko	Asymptomatic							
	Rag1-ko	Acute					Kidn, Liv, and Lun (HP, necropsy dpi 7–10)	Pulmonary hemorrhage	
	WT	Asymptomatic Renal colonization	Copenhageni strain Fiocruz L1–130 and Manilae strain L495	F	8–10	IP/2 × 10^8^	Liv, Lun, Kidn (qPCR, HP, dpi 15, 30, 60, 90, 180)	Renal fibrosis model Chronic infection	Fanton d’Andon et al. (36)
	WT	Asymptomatic Renal colonization	Copenhageni strain Fiocruz L1–130	ND	3–4	IP/10^6^	Bl and Kidn (qPCR, HP, dpi 14, 90)	Renal fibrosis model Chronic infection	Ferrer et al. (37)
	Albino	Asymptomatic Renal colonization	Manilae strain L495 (bioluminescent)	F	7–10	IP/10^7^, 10^8^	Live imaging (dpi 1–142)	Model of biphasic leptospirosis Chronic infection	Ratet et al. (38)
C3H	C3H/HeJ (*tlr4* point mutation)	Acute	Icterohaemorrhagiae	ND	3	ND/~10^7^	Lun, Kidn (HP, dpi 14, 20, 180)	Pulmonary hemorrhage Defects in CD4+ and CD8+ T cells correlate with disease progression	Pereira et al. (39)
	C3H/HeJ and C3H/HeJ/SCID	Sublethal and acute	Copenhageni strain RJ16441	ND	3–6	IP/10^6^	Liv, Kidn (HP, dpi 3–17)	No lung hemorrhage	Nally et al. (40)
	C3H/HeJ (*tlr4* point mutation)	Sublethal	Copenhageni strain Fiocruz L1-130	F	8–16	IP/10^6^, 10^7^	Uri, BL, Kidn (qPCR, HP, dpi 15)	Kidney inflammation Increased CD4+ effector T cells in spleen	Richer et al. (41)
OF1	WT	Asymptomatic	*L. borgpetersenii* Ballum strain B3-13S	ND	6–8	IP/10^8^	Kidn, Lun, Liv (HP, qPCR, dpi 14, 21, 28)	Renal carriage No nephritis	Matsui et al. (42)

*ND, not described; F, female; w.o., weeks old; IP, intraperitoneal; HP, histopathology; IF, immunofluorescence; BL, blood; Kidn, kidney; Liv, liver; Lun, lungs; Uri, urine; dpi, day post-infection*.

*Highlighted in gray are the mouse models leading to acute leptospirosis*.

### Mouse Models of Lethal Leptospirosis

As the use of inbred strains of mice became ubiquitous in laboratory testing, adult BALB/c mice were infected in the mid-70s with several genospecies of pathogenic *Leptospira* sp. These mice presented with non-lethal dissemination of the bacteria in blood and in tissues, and thus, this mouse strain was associated with resistance to acute *Leptospira* sp. infection (38). However, there are a number of examples of lethal infection of several strains of mice such as infection of adult C57BL/6 mice with 10^8^ *L. interrogans* serovar Manilae (38), infection of immunosuppressed BALB/c mice with *L. interrogans* serovar Pomona (43). Adult C57BL/6 mice deficient for toll-like receptor 4 (TLR4) or myeloid differentiation factor 88 (MyD88) (44), μMT mice devoid of B cells (44) as well as C57BL/6 mice deficient for the decay-accelerating factor (DAF)-1ko (37), were shown to die after infection with *L. interrogans* serovar Copenhageni. Furthermore, infection of 4-week old C3H–HeJ mice with *L. interrogans* serovar Icterohaemorrhagiae led to lethality with pulmonary hemorrhage (39). C3H/SCID mice devoid of B and T cells were also sensitive and died from the infection with *L. interrogans* serovar Copenhageni, without lung hemorrhage (40). Those murine models presenting targeted deficiencies in immune system receptors have been instrumental in the discovery of many key factors required to control *Leptospira* sp. in mammalian hosts and are discussed in part 2 of this review. Lethal infection of mice is observed using high doses of inoculum of *Leptospira* sp. (usually 10^6^–10^8^), whereas in the hamster model, lethal doses of inoculum of *Leptospira* sp. range between 10^2^ and 10^3^. However, infection of 5-week-old hamsters with doses ranging from 10^2^ to 10^6^ led to a progressive increase in animal survival with a 50% survival rate in hamsters infected with 10^6^ *Leptospira kirschneri* (45), which constitutes an interesting paradox. Although *Leptospira* sp. numbers such as 10^2^–5 × 10^3^ were previously quantified in urban slum water gutters in the Peruvian Amazon region of Iquitos (9), the minimum or median *Leptospira* sp. infectious dose in humans is unknown.

### Mouse Models of Sublethal Leptospirosis

Lethal outcome inconsistencies in the former studies raised questions regarding the potential usefulness of mice in understanding pathogenesis and clinical disease progression. In the last decade, there has been a surge in the number of studies investigating outcomes of experimental leptospirosis among different strains of mice such as A, CBA, BALB/c, C57BL/6, and C3H–HeJ mice, all showing sublethal infections after intraperitoneal inoculation (34–36, 41, 46) (Table 1). Infection of mice strains A, CBA, and C57BL/6 with *L. interrogans* serovar Copenhageni strain Cop led to dissemination of *Leptospira* sp. to the kidneys one month post-infection and to nephritis without apparent colonization. In BALB/c mice, no kidney lesions were observed confirming that this strain is more resistant to infection (34). Two models of sublethal leptospirosis using adult mice have been developed recently (38, 41). Sublethal infection of 7- to 10-week-old C57BL/6 mice with 10^6^–10^7^ of a bioluminescent version of *L. interrogans* serovar Manilae strain L495 allowed for live imaging of infected albino animals. The advantage of using live imaging is to allow quantification of live *L. interrogans*. Mice exhibited biphasic disease with a self-resolving hematogenous dissemination followed by renal colonization (38). In another study, 10-week-old C3H–HeJ mice were inoculated with 10^6^–10^7^ *L. interrogans* serovar Copenhageni Fiocruz L1-130. Infection led to bloodstream dissemination of *L. interrogans*, which was followed by urinary shedding, body weight loss, hypothermia, and colonization of the kidney by live spirochetes 2 weeks after infection. In addition, infection triggered inflammation of the kidney but not of the liver or the lung. Infection of mice with pathogenic *Leptospira* sp. seems to depend on infectious dose and on the mouse genetic background. The sensitivity of the techniques used to detect *Leptospira* sp. in the biological samples tested as well as the lack of well-established clinical scores such as weight loss and temperature to record disease course may account for the inconsistencies reported in the literature. As an example, long-term colonization of kidney was also observed in BALB/c mice infected with 10^7^ of the bioluminescent version of *L. interrogans* serovar Manilae strain L495 (38).

### Mouse and Rat Models of Chronic Leptospirosis

A few years after its discovery, more than 100 years ago (10), human leptospirosis was associated with the presence of rats and mice, identified as asymptomatic renal carriers of a live spirochetal bacterium, named “interrogans” because of its question mark’s shape (47). Yet, experimental infection and characterization of the disease in these animals is quite recent (48, 49), most probably because they are relatively resistant to acute disease and therefore were not considered as *bona fide* models of severe human leptospirosis. Nevertheless, knowledge of the biology of pathogenic *Leptospira* sp., the histopathology, and their survival in the reservoir host, as well as their transmission to other hosts is of utmost importance to better understand and counteract the infection in susceptible hosts, like humans.

Two studies characterized renal lesions in brown Wistar rat (*Rattus norvegicus*) experimentally infected through intraperitoneal route with *L. interrogans* serovar Copenhageni strain Fiocruz L1-130. At a high dose of 10^8^ bacteria, 1 month post-infection, all infected rats were asymptomatic, without any loss of weight compared to non-infected rats, and presented dense *Leptospira* colonization of renal tubules, without evidence of major inflammation. A total of 2–4 months post-infection around 70% of the rats presented renal interstitial nephritis, also observed in kidneys of 50% of captured wild rats found positive for *Leptospira* sp. culture (4, 50).

Interestingly, C57BL/6 mice intraperitoneally infected with 10^7^ of a bioluminescent version of *L. interrogans* serovar Manilae strain L495 (38) showed the same features as previously observed in rats (4). Renal colonization remained stable for the lifetime of C57BL6/J mice (38). *Leptospira* sp. persistence may be different in rats, depending on the bacteria serovar used for the infection. In Wistar rats, *L. interrogans* serovar Icterohaemorrhagiae persisted for 220 days but *L. interrogans* serovar Grippotyphosa persisted only 40 days (48) and *L. interrogans* serovar Copenhageni strain Fiocruz L1-130 persisted for 4 months (4). Another study using a different strain of the same bacteria serovar showed that shedding of *L. interrogans* serovar Copenhageni strain RJ16441 in the urine of Sprague-Dawley rats ceased 2–3 months post-infection (51). Altogether, these data suggest that some rats clear *Leptospira* sp. from the kidney. Nevertheless, the fact that both mice and rats shed *Leptospira* sp. for several months after kidney colonization is clearly established. The mechanism leading to the sterilization of the kidney is extremely important and remains to be investigated.

Another common feature of the rat and mouse models is a threshold of infection required to get renal colonization. In rats, the dose of 10^4^ bacteria injected intraperitoneally allows for the renal colonization of 50% of rats (4). In C57BL/6 mice, the lower limit to obtain 100% of renal colonization is 10^6^ bacteria (36, 38), but this threshold depends on the *Leptospira* sp. serovar and strain, since intraperitoneal injection with 10^3^ *L. interrogans* serovar Copenhageni strain Cop is enough to colonize the kidneys of mice (34). Given that the rat can shed up to 10^7^ *Leptospira* sp. per ml of urine (52), we may speculate that high concentration of *Leptospira* sp. in water may associate with higher transmission rates. However, the reverse may not necessarily be true.

Renal fibrosis, usually associated with inflammation, is characterized by the pathological accumulation of extracellular matrix components, such as collagen, and may compromise the kidney function of patients with leptospirosis (53). Fibrosis has also been observed in some wild rats (50) and dogs naturally infected with *L. interrogans* (54), and more recently in surviving hamsters experimentally infected with *Leptospira borgpetersenii* serovar Ballum (42). Two recent studies found that mild fibrosis occurs in kidneys of C57BL/6 mice infected with *L. interrogans* serovar Copenhageni strain Fiocruz L1-130 and serovar Manilae strain L495 (36, 37). Both studies found interstitial nephritis in infected mice 2 weeks post-infection, decreasing therafter, and sustained fibrosis from 2 weeks until 3 or 6 months post-infection. The use of antibiotic showed that fibrosis is associated with the presence of live bacteria colonizing the kidneys and not antigens (36), as previously suggested *in vitro* (55). Hence, antibiotic therapy initiated in mice 1 day after infection allowed for sterilization of kidneys, and fibrosis was not observed 15 days post-infection. However, when antibiotic treatment started 3 days post-infection, it failed to eradicate *Leptospira* sp. and fibrosis was still observed, although the kidney presented only minimal inflammation (38). In mice, it was also shown that the lack of DAF-1, an early regulator of complement cascades, aggravates fibrosis induced by infection with *L. interrogans* serovar Copenhageni strain Fiocruz L1-130 (37). Of note, the bacterial load in the kidneys of hamsters, OF1 mice (42), and C57BL/6 mice (36, 37) at the chronic phase of leptospirosis does not correlate with the extent of fibrosis, suggesting that the initial insult of penetration in the tubule, rather than colonization itself or the ensuing inflammation, is important for establishment of fibrosis.

Antibiotics administered at the chronic phase, when *Leptospira* sp. are established in the renal tubules, do not easily eradicate the pathogen in mice (19, 38) or in humans (2). In the chronic stage, Wharthin–Starry staining of rats’ kidneys has shown very dense colonization of proximal tubules by *L. interrogans* (50). Whether *Leptospira* sp. form biofilm in the tubules, as *in vitro* (56), remains to be demonstrated but it would explain the *Leptospira* sp. resistance to antibiotics in this phase of infection, in contrast to the acute phase, when antibiotics are effective if administered early (38). Interestingly, in mice, several rounds of the same antibiotic treatment at the chronic phase of disease resulted in stepwise reductions of *Leptospira* sp. (38). Together these studies suggest that a small number of bacteria reach a low number of renal tubules, where they multiply until they completely fill their niche, between 15 days and 3 weeks post-infection. Once the niche is filled, the multiplication rate would compensate the shedding, potentially explaining the observed sustained level of colonization (38). Altogether, these data suggest that the establishment of *Leptospira* sp. in the renal proximal tubules is an early event, occurring in the very first days post-infection, only when the number of *Leptospira* sp. is high enough to overwhelm the natural blood defenses. These data also imply that after the initial entry in a tubule, *Leptospira* sp. do not colonize new tubules, in mice (38). This phenomenon could be due to the efficient immunoglobulin (Ig) response (44, 47) that would control any *Leptospira* sp. leaving a tubule to go back into circulation, before it may infect another nephron. Only two studies in the 1980s addressed the mechanism of entry of the *Leptospira* sp. in the tubules, using experimentally infected swine with *L. interrogans* serovar Pomona, or mice. They described a biphasic infection with first a hematogenous dissemination of *Leptospira* sp. in the kidney, followed in the first 4 days post-infection by the tubular phase, where *Leptospira* sp. cross the tubules till the lumen [reviewed in Ref. (52)]. Thereafter, the proximal tubule of the kidney constitutes a safe niche where *Leptospira* sp. are protected from the activity of the immune system and do not cause major lesions, aside from mild fibrosis, a reminder of the initial tubular insult. The reason why *Leptospira* sp. localizes in the proximal tubules is unknown.

Since chronic asymptomatic leptospirosis with prolonged shedding of *Leptospira* sp. in the urine has also been observed in humans (9, 57), these results should emphasize the fact that prophylactic and antibiotic treatments in humans, like serum therapy (58), are important to be administered early after infection to avoid renal colonization that in the long term may weaken the kidney (59).

Although the intraperitoneal route of infection has been widely used in leptospirosis animal models, it may not reflect the whole process of infection since *Leptospira* sp. penetrate the body through abraded skin or mucosa. A recent study compared infection with 10^7^ *L. interrogans* serovar Copenhageni strain Fiocruz L1-130 in 7-week-old Wistar rats through mucosal, subcutaneous and intraperitoneal routes. Rats infected through all routes remained largely asymptomatic. One month post-infection, kidneys of all rats infected intraperitoneally were colonized, without any sign of interstitial nephritis, although only five out of eight rats infected through the mucosal route and only one out of eight rats infected subcutaneously were colonized (60), suggesting that natural infection does not systematically lead to renal colonization in 7-week-old Wistar rats.

Mouse models of sublethal and chronic infection may allow us to better understand leptospirosis and host factors that lead to immune evasion, which can result in acute or chronic disease and susceptibility or resistance to infection (41).

## Profiling Immune Responses to Pathogenic *Leptospira* Using Mouse Models

The wide availability of genetically defined congenic strains of mice is currently leading to a new trove of knowledge on the engagement of immune system components with pathogenic *Leptospira* sp., which informs our understanding of the pathways to, and the markers for, protective immunity (61).

### Protective Role of Antibodies against Lipopolysaccharide (LPS) and Roles of TLR4 and TLR2

The humoral response against pathogenic *Leptospira* sp., described as a “bactericidal substance for the spirochaetae in the blood of patients” and its ability to destroy *Leptospira* sp. has been demonstrated since 1916. In those studies, serum from immunized horses and goats or from convalescent patients with leptospirosis was administered to guinea pigs experimentally infected with *Leptospira* sp. or to leptospirosis patients, respectively (10, 58). The authors drew very robust conclusions about their data. Indeed, to test the efficiency of sera to clear *Leptospira* sp. from the blood of infected animals or patients suffering from leptospirosis, they injected the blood, with or without *Leptospira* sp., into naïve guinea pigs that subsequently did or did not develop leptospirosis (58). More recently, mostly mouse models of leptospirosis produced important clues about the immune cells and receptors involved in susceptibility to leptospirosis (40, 43, 44, 62, 63) (highlighted in Table 2). Not surprisingly, B cells appeared to be key players and are important to control leptospirosis. Indeed, BALB/c mice chemically depleted of B cells were shown to be susceptible to lethal leptospirosis induced by *L. interrogans* serovar Pomona in contrast to untreated WT mice (43). This observation was confirmed using C3H/SCID mice and μMT mice on a C57BL6/J background, both genetically deficient for B cells. These mice also died from acute leptospirosis when infected with *L. interrogans* serovar Copenhageni (40, 44). Interestingly, the antibody response to *Leptospira* sp. has been shown to appear quickly after infection (58), as early as 3 days in the case of IgM after intraperitoneal infection with *L. interrogans* serovar Copenhageni (43, 44).

**Table 2:** **Mice as animal models to study innate and adaptive immune responses to *Leptospira* sp. infection**.

Mouse strain	Genotype	Model of disease	Pathogenic *Leptospira* sp. serovar and strain	Biological variables			Tissue dissemination (technique, dpi)	Major findings	Reference
				Sex	Age (w.o.)	Infection route/dose			
BALB/c	Wild type (WT)	Asymptomatic	Pomona and Hardjo	M, F	~6	IP/~10^8^ 10^9^	BL (dpi 1–30)	B cells are important to control *Leptospirosis*	Adler and Faine (43)
	Immunosuppressed	Acute							
	Athymic nude	Asymptomatic	Pomona L10 and Copenhageni L45	ND	5–6	IP/~3 × 10^8^	BL (dpi 2–10)	Humoral response crucial to control *Leptospirosis*	Adler and Faine (64)
	Immunosuppressed	Acute							
	WT	Asymptomatic	Copenhageni strain Fiocruz L1-130	ND	ND	IP/2 × 10^8^	Kidn (HP dpi 28)	No interstitial nephritis	Athanazio et al. (46)
	IL4ko	Asymptomatic							
	WT	Asymptomatic	Icterohaemorrhagiae strain Cop	F	4–5	IP/10^6^	BL, Kidn (IF dpi 28)	High IgG response No kidney lesions	Santos et al. (34)
C57BL/6	WT, TNFRko, IFNγko	Asymptomatic	Copenhageni strain Fiocruz L1-130	ND	ND	IP/2 × 10^8^	Kidn (HP dpi 28)	TNFR involved in interstitial nephritis	Athanazio et al. (46)
	WT, CD3ko, TLR2ko	Asymptomatic	Copenhageni strain Fiocruz L1–130	F	8–10	IP/2 × 10^8^	BL, Kidn (qPCR, qRT-PCR dpi 3)	Toll-like receptor 4 (TLR4) and TLR2-dependent IgM, IgG, iNOS and IFN-γ responses	Chassin et al. (44)
	TLR4ko; TLR2/4dko; MyD88ko	Lethal							
	μMT; Rag2ko	Lethal							
	CD3ko WT	Asymptomatic Asymptomatic	Copenhageni strain Fiocruz L1-130	F	8–10	IP/10^6^	Kidn (qPCR, ELISA, qRT-PCR dpi 3)	Downregulation of transporters in kidneys NLRP3/TLR2/4 dependent IL1β secretion	Lacroix-Lamande et al. (65)
	TLR2/4dko	Sublethal infection							
	WT, TLR2ko; TLR3ko; TLR5ko; TLR9ko	Asymptomatic chronic renal colonization	Copenhageni strain Fiocruz L1–130 and Manilae strain L495	F	8–10	IP/10^6^	Liv, Lun, and Kidn (qPCR and HP dpi 15)	TLRs, NLRs, T and B-cells are not involved in fibrosis iNOS participates in fibrosis	Fanton d’Andon et al. (36)
	TLR4ko; TLR2/4dko; MyD88ko	Sublethal infection							
	Nod1ko; Nod2ko; Nod1/2dko, Casp1ko	Asymptomatic							
	μMT	Sublethal							
	iNOSko	Asymptomatic							
	CD3ko	Asymptomatic							
	Daf1ko	Lethal chronic renal colonization	Copenhageni strain Fiocruz L1–130	ND	3–4	IP/10^6^	BL and Kidn (qPCR and HP dpi 14 and 90)	Lack of Daf1 enhances nephritis and fibrosis	Ferrer et al. (37)
	WT	Asymptomatic	Copenhageni strain Fiocruz L1–130 and Manilae strain L495	M	4	IP/5 × 10^6^	BL, Kidn, and Liv (qPCR and HP dpi 3)	Pathogenic *Leptospira* sp. infection trigger formation of neutrophil extracellular traps	Scharrig et al. (66)
C3H	C3H/HeJ (*tlr4* point mutation)	Lethal and sublethal	Icterohaemorrhagiae strain HAI188	F	3–6	IP/6 × 10^8^ IP/10^8^	Heart, Spl, Kidn, Lun, and Liv (qPCR and HP dpi 21)	TLR4 response important to control *Leptospira* sp. infection	Viriyakosol et al. (63)
	C3H/SCID								
	C3H/OuJ (functional TLR4)	Asymptomatic							
	C3H/HeJ	Lethal and sublethal	Copenhageni strain Fiocruz L1-130	ND	3	IP/10^7^	Kidn (qPCR and HP dpi 14)	Protective role of NO against *Leptospira* sp.	Pretre et al. (67)

*dko, double knockout; ND, not described; F, female; M, male; w.o., weeks old; IP, intraperitoneal; BL, blood; Kidn, kidney; Liv, liver; Lun, lungs; Spl, spleen; HP, histopathology; IF, immunofluorescence; dpi, day post-infection*.

*Highlighted in blue are the mouse models potentially leading to lethal infection*.

It is known that most of the protective antibody response to *Leptospira* sp. infection are directed against the LPS. However, *Leptospira* sp. LPS is different from one serovar to another, which undermines serovar cross-protection (68). Definite evidence was provided in 1986 with a monoclonal antibody elicited against LPS that protected guinea pigs against leptospirosis (69). A more recent study confirmed passive immunization of guinea pigs with agglutinating monoclonal antibodies against LPS (70). The fact that Nude mice, unable to produce T cells, were still able to mount a protective immune response suggested that protection was T cell independent (64), which was consistent with the fact that LPS is the target of the antibodies since this molecule is known to mediate T independent responses. This observation was confirmed using CD3ko mice, lacking T cells (44). The crucial humoral response is mediated by TLR4, a member of the toll-like receptor family of innate receptors. Those receptors are involved in microbial recognition, through conserved molecular patterns, such as LPS, and are involved in the expression of antimicrobial peptides, chemokines, cytokines, and molecules of costimulation, leading to recruitment of immune cells, which culminates in the elimination of the pathogen, and therefore protection. Indeed, C3H/HeJ mice, known for decades to be resistant to LPS endotoxin shock, were often used as model of infection since they are more susceptible to many different pathogens (i.e., *Borrelia burgdorferi*). They were subsequently shown to have a mutation in TLR4 (71). Four-week-old C3H/HeJ are sensitive to acute leptospirosis upon infection with *L. interrogans* serovar Icterohaemorraghiae (39). Later on, C3H/HeN mice that do not have the point mutation in TLR4 were infected in parallel with C3H/HeJ mice, and proven resistant to death, providing further evidence that TLR4 is important to control leptospirosis (63). One of the mechanisms of susceptibility linked to TLR4 has been shown to rely on the early production of IgM directed against the LPS, and on the production of IgG relying on both TLR4 and TLR2 (44). The leptospiral LPS recognition by TLR4 is most probably the major factor influencing the susceptibility or resistance to infection and disease progression, since the lack of this receptor is enough to confer susceptibility to *Leptospira* sp. infection (44). LPS from *L. interrogans* is atypical and is not recognized by TLR4 in human cells, whereas mice that are able to recognize the *Leptospira* sp. LPS are resistant to lethal leptospirosis (72, 73), providing an explanation why humans are sensitive to acute leptospirosis. Mice are therefore excellent reservoir hosts in the enzootic cycle that maintains this pathogen in the environment. It is tempting to speculate that TLR4 from hamsters, gerbils, and guinea pig would also not be able to recognize the *Leptospira* sp. LPS. Recently, a study using mice showed that TRIF, the adaptor of TLR4 and also TLR3, the receptor of viral RNA, has a protective role during leptospirosis (74). However, the role of TRIF has not been further studied (74), but the results showing decreased humoral defense or higher *L. interrogans* burden in the organs of TRIFko mice suggest that the role of TRIF is indeed linked to TLR4. Also, LPS from *Leptospira* sp. is atypically recognized by TLR2, the receptor of lipoproteins, and this is not conferred by the lipid A moiety, but most probably by a lipopeptide linked to the O antigen (72, 73), which is still unknown. Hence, intraperitoneal injection of purified LPS from *L. interrogans* serovar Icterohaemorrhagiae strain Verdun is able to kill C57BL6/J WT mice but not TLR2ko mice, in a model of LPS toxicity leading to liver injury and mortality, after sensitization of mice with d-galactosamine and IFNγ (72). Whether this atypical TLR2 reactivity of the LPS is linked to virulence is unknown.

LipL32, the major outer membrane (OM) lipoprotein of *Leptospira* sp. is recognized by TLR2, and therefore is able to induce inflammation *in vitro* in proximal kidney cells (72, 75, 76). However, the relevance for kidney infection is not clear, since the lack of TLR2 did not change fibrosis and did not reduce inflammation in kidneys of mice 15 days post-infection with *L. interrogans* (36). Fibrosis was also induced only in the presence of live bacteria, not in the presence of LipL32 antigens, present after an antibiotic treatment (36). Nevertheless, *Leptospira* sp. antigens from the OM, including the lipoprotein LipL32 may be responsible for nephritis observed in other animals, as was very elegantly demonstrated in zebrafish larvae (77). Posttranslational modifications of LipL32 have recently been studied using *Leptospira* sp. retrieved from urine of rats chronically infected with *L. interrogans* serovar Copenhageni strain RJ16441 (78). The authors showed acetylation or trimethylation of lysines of LipL32, only in the case of *Leptospira* sp. retrieved from urine but not from *Leptospira* sp. grown in culture. They further showed that the lysine modifications lowered the reactivity toward sera from infected patients, suggesting a role in escape from the immune response and helping the maintenance of *Leptospira* sp. in the proximal tubules. Of note, the peculiar *Leptospira* sp. lipid A was also shown to be methylated, the methyl group inactivating a phosphate group (79), known to be important for human TLR4 recognition of lipid A. Whether posttranslational methylation modification is a general strategy of *Leptospira* sp. to escape the immune response remains to be investigated.

### Protective Host Mediators in Leptospirosis

IL-1β is a pleiotropic cytokine, central in inflammation. Its expression is tightly regulated by two signals, the first deriving from a NF-κB pathway such as TLR or TNF stimulation. The second signal activates the inflammasome, a platform of proteins leading to activation of Caspase 1, able to cleave the pro-IL-1β to mature IL-1β, which can be secreted. Using mouse bone marrow-derived macrophages *in vitro*, it has been shown that *Leptospira* sp. trigger these cells to produce IL-1β, through the Nod-like receptor protein 3 (NLRP3) inflammasome (65). In mice, LPS and lipoproteins are recognized by TLR4 and TLR2, which participate in IL-1β secretion by priming mRNA expression of the pro-IL-1β and NLRP3. *Leptospira* sp. activate the NLRP3 inflammasome through a dysregulation of the potassium flux (65), due to glycolipoprotein action, known to downregulate the potassium pump (80). This has been confirmed *in vivo* after infection with *L. interrogans* serovar Copenhageni strain Fiocruz L1-130 of TLR2ko or TLR4ko mice. It was shown that all the transporters were downregulated and the IL1β decreased in both TLR2ko and TLR4ko mice, 3 days post-infection (65).

Another inflammatory cytokine, IFN-γ is generally recognized as a protective cytokine, able to prime the phagocytic activity of macrophages. IFN-γ mRNA expression peaks in hamsters between 8 and 18 h post intraperitoneal infection with *L. interrogans* serovar Icterohaemorraghiae strain Verdun in blood (24). Upon infection with *L. interrogans* serovar Copenhageni strain Fiocruz L1-130, IFN-γ mRNA is expressed in the organs of mice 3 days post-infection through a TLR2- and TLR4-dependent pathway (44). IFN-γ has been shown to be produced by T cells in kidney and also by parenchymal cells, and in liver, its production is linked to the presence of B cells (44). The beneficial effect of T cells, potentially through IFN-γ production, has been shown in the lungs and kidneys of C3H/HeJ mice infected with *Leptospira* sp. and depleted in CD4 and CD8 T cells (65) and in CD3ko/C57BL/6 mice (44).

Nitric oxide (NO) is an antimicrobial compound, produced by the inducible nitric oxide synthase (iNOS) in macrophages and endothelial cells. Upon infection with *Leptospira* sp., iNOS is expressed 3 days post-infection in kidneys and lungs of mice in a TLR2- and TLR4-dependent manner (44). NO is secreted by parenchymal cells in kidneys of infected mice (44), and it plays a damaging role in nephritis (35) and in renal fibrotic lesions induced by *Leptospira* sp. (36). Experiments conducted with both Syrian hamsters and C3H/HeJ mice treated with a specific inhibitor of iNOS showed increased mortality and aggravated renal lesions upon infection with *L. interrogans* serovar Copenhageni strain Fiocruz L1-130, suggesting that overall NO plays an important antimicrobial role against *Leptospira* sp. (67). Of note, in renal lesions and in pulmonary hemorrhage induced by *L. interrogans* serovar Copenhageni, the potential contribution of autoimmune response has been ruled out since in both cases the lesions were still present in mice without B and T cells (35, 36).

Other than protective responses resulting from the stimulation of both TLR4 and TLR2, it was shown that leptospirosis induced a TLR2- and TLR4-independent inflammation, also independent from other TLRs. Indeed, in mice deficient for MyD88, the adaptor of almost all TLRs except TLR3, the inflammation upon infection with *L. interrogans* was equivalent to the inflammation found in double TLR2/TLR4ko mice (44). The receptor responsible for this detrimental inflammation remains to be determined.

### Role of Neutrophils and Macrophages in Leptospirosis

Although neutrophilia is a common feature of human leptospirosis, only few neutrophils were identified in kidneys of mice 3 days post-infection with *L. interrogans* (44, 66) and depletion of neutrophils did not change the overall course of the disease (44, 66, 81), suggesting that neutrophils are not major players in murine leptospirosis. However, a recent study in C57BL/6 mice showed that *L. interrogans* serovar Copenhageni strain Fiocruz L1-130 triggered, in the dissemination phase, the generation of neutrophil extracellular DNA traps (66). NETs from human neutrophils killed *L. interrogans in vitro*. The depletion of neutrophils in mice resulted in increased bacteria 3 days post-infection in blood and also in kidneys 14 days post-infection, although nephritis was not changed, compared to untreated infected mice. These results suggest that NETs are an important mechanism of host defense occurring early, impairing the dissemination of bacteria in the tissues. However, *Leptospira* sp. that manage to settle in the kidneys are obviously able to escape this defense (66), suggesting that these bacteria reached the kidneys before the onset of NET generation 1–2 days post-infection. Alternatively, NET escape may be due to a nuclease activity found in pathogenic but not in saprophytic *Leptospira* sp. (66). Unchanged nephritis despite higher bacterial colonization is difficult to interpret but reminds us that fibrosis is not proportional to the bacterial load (36).

Several *in vitro* and one *in vivo* study depleting macrophages with silica particles suggest that macrophages can phagocyte *Leptospira* sp., at least when they are opsonized with specific Ig (43, 64, 82). This has been recently confirmed *in vivo* using zebrafish (83). Indeed, zebrafish embryos are a powerful model to study host–pathogen interactions. They have a functional innate immune system close to the mammalian immune system. The embryos are transparent and allow tracking of fluorescent bacteria. Using Syto-83 dye labeled and unstained *L. interrogans* serovar Copenhageni strain Fiocruz L1-130, the authors showed rapid encounter and internalization of *Leptospira* sp. in phagocytes 2 h post intravenous injection and survival of *Leptospira* sp. in these cells until 48 h post-infection (83). Strikingly, the infected phagocytes changed morphology and specifically migrated in a dorsal part of the embryo, which has never been observed with other pathogens, suggesting that phagocytes could help *Leptospira* sp. traffic inside the host (83). Whether this observation could be relevant in mammals is unknown.

### Human Genetic Polymorphisms Associated with Susceptibility to Leptospirosis

Human genetic polymorphism studies are very useful to understand the mechanisms involved in immune response to diseases. Only a few studies have been performed on polymorphisms in innate immune genes known to be associated with infectious diseases that could be relevant for leptospirosis (84–86). A study on an Argentinian population suggested that polymorphisms on both *tlr2* and in *tlr1* genes may confer enhanced susceptibility to severe leptospirosis (86). It has also been suggested that, among other factors, polymorphisms in *IL-4* and *IL-4R* (84) and *IL-1*β, *IL-12R*, and *CISH* (multiple cytokines inducible SH2-containing protein gene) (85) correlated with susceptibility to leptospirosis. However, these studies produced discordant results, despite the fact that two were performed a few years apart in two distinct cohorts of patients from Terceira (84) and São Miguel (85) neighboring islands in the Azores archipelago, Portugal. The role of IL-4 has been studied in mice, but the resistance of BALB/c/IL-4ko mice to the infection with *L. interrogans* serovar Copenhageni strain Fiocruz L1-130 was comparable to WT BALB/c, suggesting that IL4 does not play a major role in leptospirosis in these mice (46). The association of *tlr2* polymorphism with leptospirosis (86) was not found in the Terceira Island study (84), which also did not find the *IL-1*β polymorphism found in the São Miguel Island (85). Interestingly, the São Miguel study did not find polymorphisms in TLR4 associated with susceptibility to leptospirosis (85), which is consistent with the finding that the atypical LPS from *Leptospira* sp. does not signal through TLR4 in humans (69, 70). The small cohorts (around 100) of leptospirosis patients may be one of the factors decreasing the significance of these results. Thus, larger studies with multifactorial analysis are needed to confirm the potential protective roles of cytokines and TLRs in human leptospirosis.

**Figure 1:**
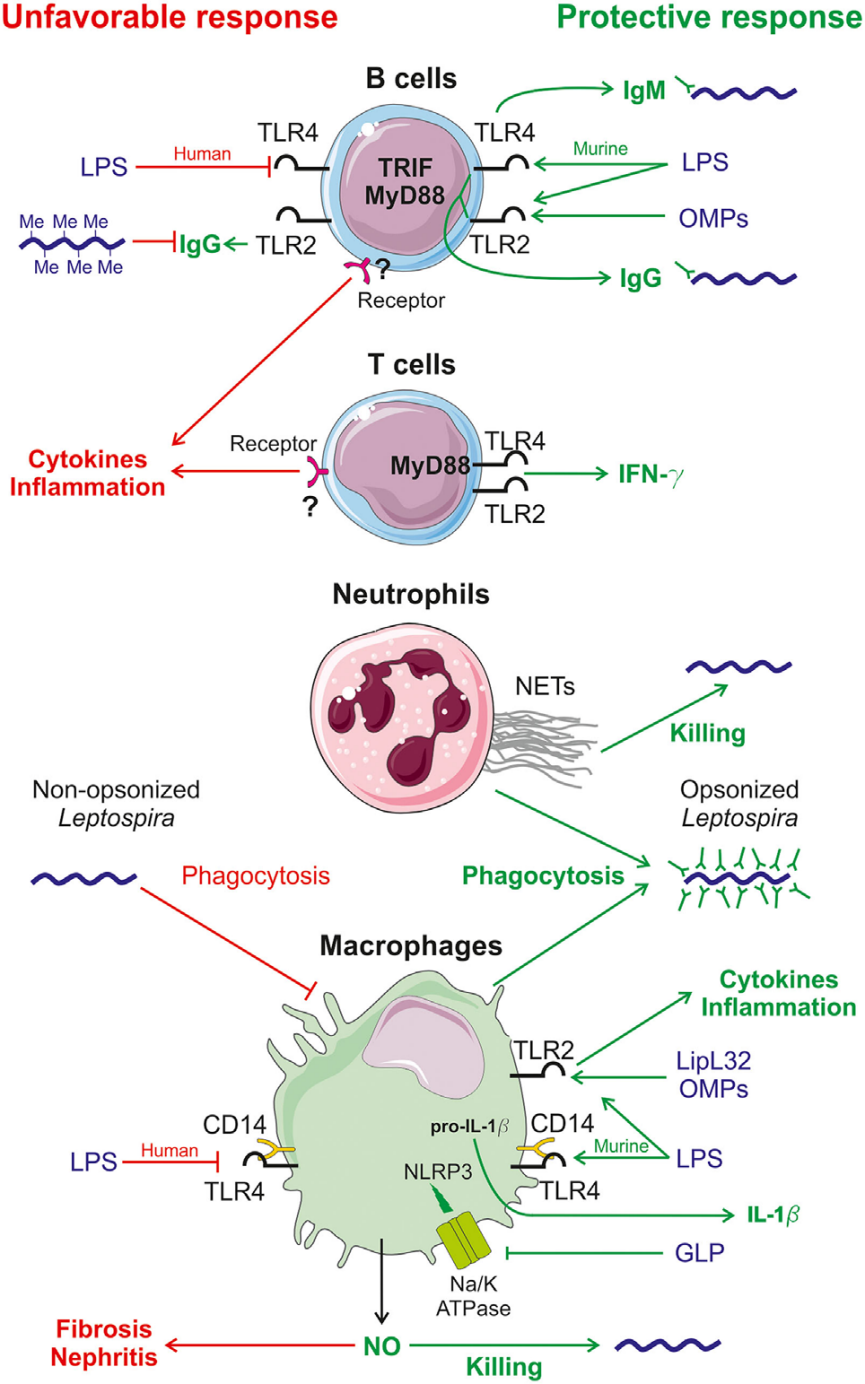
**Diagram of immune responses induced by *Leptospira* sp. infection in mice**. Known innate responses to *Leptospira* sp. involve neutrophils, macrophages but also B and T cells. Recognition of *Leptospira* sp. mostly occurs through the TLR2 pathway, sensing outer membrane proteins (OMPs) such as the lipoprotein LipL32, the major leptospiral OMP and the atypical LPS (72), which is also recognized by toll-like receptor 4 (TLR4) in mice (73). Protective host responses are depicted in green on the right side, whereas potential unfavorable responses are in red, on the left side. In mouse B cells, TLR4 stimulation by leptospiral LPS leads to the early production of partially protective IgM (44), via the TRIF adaptor (74). TLR2 and TLR4 responses, through the Myd88 adaptor, also control the production of protective IgG (44). *In vivo* methylation of LipL32 in rat has been shown to reduce its recognition by human antiserum (78). In humans, leptospiral LPS is not recognized by TLR4 (73), potentially leading to disease, as observed with TLR4 mutant mice (39, 44, 63). In mouse T cells, *Leptospira interrogans* signal through the MyD88-dependent activation of TLR4 and TLR2 receptors and trigger the production of the protective pro-inflammatory cytokine IFN-γ that activates macrophages (44). Both T and B cells, by sensing leptospiral components through an unknown receptor, are involved in the production of an unfavorable pro-inflammatory cytokine response (44). In humans, neutrophils play a slight protective role for the host against *Leptospira* sp. infection due to the production of bactericidal neutrophil extracellular traps (NETs) (66). Neutrophils and macrophages barely phagocytize non-opsonized *Leptospira* sp. but opsonized *Leptospira* sp. with specific IgG are readily killed by phagocytose (43, 82). Macrophages produce pro-inflammatory cytokines leading to a protective inflammatory state by sensing OMPs, including LipL32, via TLR2 (72). The atypical leptospiral LPS is also detected by TLR2 and CD14, the co-receptor of TLR4 (72). Acting in concert with TLR2 and TLR4 activation, leptospiral glycoprotein blocks the Na/K-ATPase pump that triggers the activation of the Nod-like receptor protein 3 (NLRP3) inflammasome and enables the secretion of the pro-inflammatory cytokine IL-1β (65). Upon *L. interrogans* infection, macrophages and other cells also produce nitric oxide (NO), which has a positive and a negative effect. NO has a protective effect through the action of its antimicrobial role (67) and the negative effect is that NO activity favors kidney fibrosis (36) and nephritis of infected hosts (35, 67).

A summary of the use of mice as animal models to study innate and adaptive immune responses to *Leptospira* sp. infection is provided in Table 2, and a diagram of the immune response to *Leptospira* sp. infection is provided in Figure 1.

## Virulence Factors of *Leptospira* sp. Assessed with Animal Models of Leptospirosis

To establish whether a gene encoded by a pathogen can contribute to its virulence, the classical approach involves genetic inactivation and study of the outcome of infection with the mutant strain. Targeted genetic manipulation of *Leptospira* sp. is still not achievable on a routine basis; however, random mutagenesis by transposon insertion made it possible to generate mutant strains that can be tested in animal models to evaluate the role of the mutated gene in virulence. In a limited number of studies, complementation of the mutant was achieved (87–90) and allowed to fulfill the molecular Koch’s postulates (91), since the complementation of the mutated non-virulent (NV) strains restored virulence.

**Figure 2:**
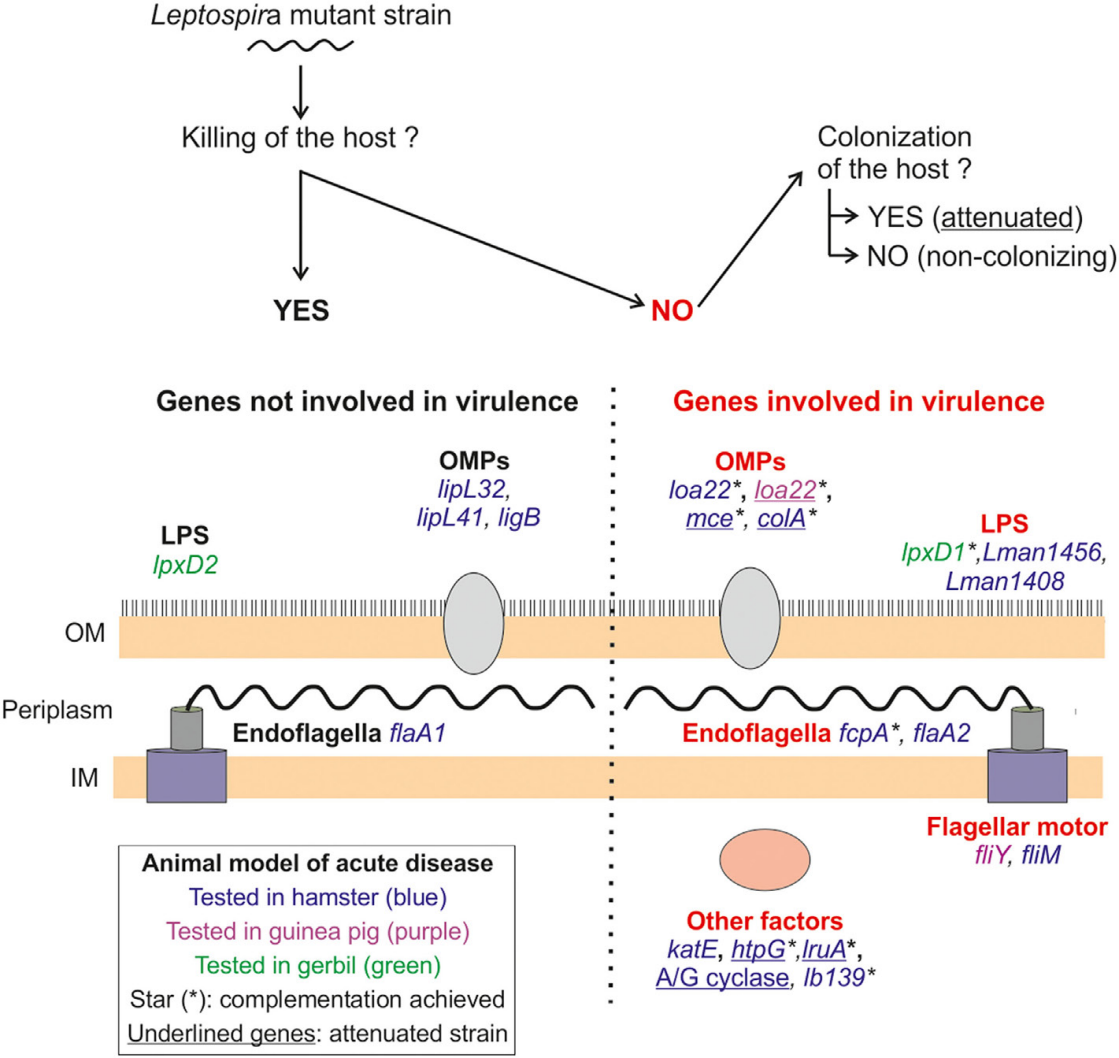
***Leptospira* sp. virulence factors identified in animal models of lethal disease**. The panel depicts the different factors (in capitals) and genes tested for virulence in different animal models (summarized in Tables 3 and 4) and their localization in *Leptospira* sp. The mutant strains were inoculated via the intraperitoneal route, using both lethal and sublethal bacterial doses, in different animal models for acute disease: hamster (blue), guinea pig (purple), and gerbil (green). In all cases, the mutant strains were compared with their wild-type counterpart to assess the effect on virulence of the mutated genes. As schematized on top of the panel, we discriminate the genes in two groups; the genes not involved in virulence (on the left side of the panel), since the mutant strain killed the host, and the virulence genes (right side of the panel) determined since the mutant strain did not cause the death of animal. In this category, we further distinguish the non-virulent (NV) mutants that did not colonize target organs and the NV attenuated ones that did. Attenuated mutants are indicated by underlined names. Complementation of mutated genes was not always achieved, and the complemented mutants are indicated with a star (*). OM, outer membrane; IM, inner membrane; OMP, outer membrane protein.

This section describes the *Leptospira* sp. virulence factors characterized in studies fulfilling the following criteria: (1) no difference in the *in vitro* growth rate at 30°C between the WT and mutant strains; (2) the WT *Leptospira* sp. strain caused death of all the infected individuals (lethal challenge), whereas the mutant strain, at equivalent dose, results in the survival of all animals. Moreover, we discriminate the bacterial phenotype as V, causing death of the host, or NV, which does not kill the host and is not found in organs and kidneys (Figure 2). A mutant strain is considered NV attenuated when it still colonizes target organs, including kidneys. Hamsters, guinea pigs, and gerbils used for studying acute leptospirosis will succumb to the infection in a period ranging from 5 to 10 days for V strains, whereas they will survive with no clinical signs of the infection when NV or attenuated strains are administrated. Some of the studies also included a test in rats or mice to check kidney colonization.

As shown in Figure 2 and Tables 3 and 4, around 20 genes have been tested for their potential role in virulence in leptospirosis. Half of them have been identified as essential for virulence (highlighted in Tables 3 and 4). Two components of the cell wall, the endoflagella and LPS, appear to be true virulence factors, since mutations in several genes involved in motility or LPS synthesis, result in the loss of virulence (Figure 2; Table 3).

**Table 3:** **Animal models to study *Leptospira interrogans* mutants in motility and LPS biosynthesis genes**.

Function	*L. interrogans* serovar and strain	Gene/name	Biological variables				Bacterial phenotype	Tissue dissemination (technique, dpi)	Reference
			Animal	Sex	Age (w.o.)	Infection route/dose			
Motility	Australis *702*	*fliM* (LIC11836) Flagellar motor switch protein	Hamster	F	6	IP/10^6^, 10^8^	NV (**c^+^**)	ND (dpi 21)	Fontana et al. (89)
	Manilae strain L495	*flaA1* (LIC10787) Flagellar subunit	Hamster	M	4	IP/10^3^, 10^6^	V	Kidn+; Liv+ (qPCR, dpi 5–7)	Lambert et al. (92)
	Manilae strain L495	*flaA2* (LIC10788) Flagellar subunit	Hamster	M	4	IP/10^3^, 10^6^	NV	Kidn−; Liv− (qPCR, dpi 21)	Lambert et al. (92)
	Lai strain Lai	*fliY* (LA2613) Flagellar motor switch protein	Guinea pig	ND	3	IP/6 × 10^8^, 1.2 × 10^9^, 3 × 10^9^, 6 × 10^9^	NV	ND	Liao et al. (93)
	Copenhageni strain Fiocruz LV2756	*fcpA* Flagellar coiling protein	Hamster	M	3–6	IP/10^8^	NV (**c^+^**)	Kidn−; Liv−; Lun−; Spl−; Eye−; BL− (qPCR, dpi 21)	Wunder et al. (88)
						CJ/10^8^	NV	Kidn−; Liv−; Lun−; Spl−; Eye−; BL− (qPCR, dpi 21)	
LPS synthesis	Manilae strain L495	*lpxD1* (LA051) LPS modification enzyme	Gerbil	ND	ND	IP/10^4^	NV (**c^+^**)	Kidn− (qPCR, culture, dpi 20)	Eshghi et al. (90)
	Manilae strain L495	*lpxD2* (LA4326) LPS modification enzyme	Gerbil	ND	ND	IP/10^4^	V	Kidn+ (qPCR, culture, dpi 20)	Eshghi et al. (90)
	Manilae strain L495	*Lman-1456* (LA1641) LPS synthesis	Hamster	ND	ND	IP/10^3^	NV	Kidn− (culture, dpi 21)	Murray et al. (94)
						CJ/10^6^	NV	ND	
	Manilae strain L495	*Lman-1408* (LA1641) LPS synthesis	Hamster	ND	ND	IP/10^3^	NV	Kidn− (culture, dpi 21)	Murray et al. (94)

*M, male; F, female; ND, not described; w.o., weeks old; IP, intraperitoneal; CJ, via conjunctiva; NV, non-virulent; V, virulent; **c^+^**, complementation restored virulence; BL, blood; Kidn, kidney; Liv, liver; Lun, lungs; Spl, spleen; dpi, day post-infection*.

*The numbers in brackets refer to the corresponding annotated genes in *L. interrogans* Copenhageni strain Fiocruz L1-130 (LIC), or in *L. interrogans* Icterohaemorraghiae strain Lai (LA)*.

*Highlighted in light orange are the genes involved in virulence*.

**Table 4:** **Animal models to study *Leptospira interrogans* mutants in outer membrane proteins (OMPs) genes and other factors**.

Function	*L. interrogans* serovar and strain	Gene/name	Biological variables				Bacterial phenotype	Tissue dissemination (technique, dpi)	Reference
			Animal	Sex	Age (w.o.)	Infection route/dose			
OMPs	Manilae strain L495	*lipL32* (LIC11352) OMP	Hamster	ND	ND	IP/10^3^	V	Kidn+; Lun+; Liv+ (HP, dpi 7–14)	Murray et al. (95)
				ND	ND	CJ/10^6^	V	ND	
			Wistar rat	ND	6	IP/10^8^	V	Kidn+ (HP, dpi 15)	
	Manilae strain L495 Pomona strain LT993	*lipL41* (LA0615) OMP	Hamster	M/F	4–6	IP/10^3^, 10^4^	V	Kidn+ (culture, dpi 21)	King et al. (96)
	Lai strain Lai 56601	*loa22* (LA0222) OmpA-like protein	Hamster	M	6–8	IP/5 × 10^7^, 10^8^	NV (**c^+^**)	ND	Ristow et al. (87)
			Guinea pig	M	2–3	IP/2 × 10^8^, 4 × 10^8^	NV (**c^+^**) A	Kidn+; Liv− (culture, dpi 21)	
	Lai strain Lai 56601	*colA* (LA0872) Collagenase	Hamster	M	ND	IP/10^6^	NV (**c^+^**) A	Kidn+; Lun+; Liv+; Uri+; BL+ (CFU, dpi 14)	Kassegne et al. (97)
	strain Lai	*mce* Mammalian cell entry protein	Hamster	M	4	IP/10^5^, 10^6^, 10^7^, 10^8^, 10^9^	NV (**c^+^**) A	Uri+ (CFU, dpi 14)	Zhang et al. (98)
	Copenhageni strain Fiocruz L1-130	*ligB*	Hamster	M	5–8	IP/10, 10^2^,10^4^ 10^6^	V	Kidn+	Croda et al. (99)
			Wistar rat	ND	4–5	IP/10^8^	V	Kidn+ (culture, dpi 9)	
Other factors	Manilae strain L495	LIC12327 Adenylate/guanylate cyclase	Hamster	F	4	IP/10^6^	NV A	BL+; Kidn+; Liv+ (qPCR, dpi 4)	Lourdault et al. (100)
	Manilae strain L495	LB139	Hamster	ND	ND	IP/10^6^	NV (**c^+^**)	BL−; Kidn−; Liv− (qPCR and IF dpi 5 and 25)	Eshghi et al. (101)
						CJ/10^7^	NV (**c^+^**)	ND	
	Manilae strain L495	*htpG* (LB058) High-temperature protein G	Hamster	M/F	4–6	IP/10^3^, 10^5^, 10^7^	NV (**c^+^**) A	Kidn+; Lun+; Liv+ (qPCR, dpi 5)	King et al. (102)
	Manilae strain L495	*lruA* (LIC11003)	Hamster	M	4	IP/10^3^	NV A	Kidn+ (qPCR, dpi 21)	Zhang et al. (103)
	Manilae strain L495 Pomona strain LT993	*katE* (LA1859; LIC12032) Catalase	Hamster	M	4	IP/10^6^	NV (L495)	ND (dpi 21)	Eshghi et al. (104)
							NV (LT993)	ND (dpi21)	

*M, male; F, female; ND, not described; w.o., weeks old; IP, intraperitoneal; CJ, via conjunctiva; NV, non-virulent; V, virulent; NV A, attenuated or partially virulent; **c^+^**, complementation restored virulence; BL, blood; Kidn, kidney; Liv, liver; Lun, lungs; Uri, urine; CFU: colony forming units; HP, histopathology; IF, immunofluorescence; dpi, day post-infection*.

*The numbers in brackets refer to the corresponding annotated genes in *L. interrogans* Copenhageni strain Fiocruz L1-130 (LIC), or in *L. interrogans* Icterohaemorraghiae strain Lai (LA)*.

*Highlighted in light orange are the genes involved in virulence and in light yellow the attenuated mutant strains, non virulent but still colonizing the kidneys*.

### Endoflagella As a Virulence Factor

A mutant of the flagellar subunit FlaA2 (92) resulted in a flagellum more flexible than the WT. This bacterium lost translational motility and was not virulent in hamsters. Moreover, 5 days post-infection, the FlaA2 mutant was not found in organs, suggesting that motility is also essential for early dissemination in tissues (92). Similarly, an abundant protein exposed at the surface of the flagellum filament, called Flagellar coiling protein (Fcp)A was recently characterized (88). Mutants in the *fcp*A gene, either from clinical isolates or constructed by allelic exchange, lost the hook shape, presented uncoiled flagella, and lost motility (88). This mutant also lost its ability to translocate *in vitro* across polarized kidney cells (88), suggesting that the step of active penetration in the tubule might require bacterial motility. Moreover, the intraperitoneal or conjunctival infection of FcpA mutant strains in hamsters did not result in disease or colonization of the kidneys, although complementation restored the flagella coiling, translational motility, and virulence. Mutations on different components of the flagellar motor switch, such as FliY (93) and FliM (89), resulted in mutant strains that are deficient in motility in soft agar plates and were not able to cause disease in guinea pigs or hamsters, respectively. Of note, the trans-complementation with a WT copy of the *fli*M gene on the pMaori replicative plasmid (105) restored motility and virulence. These results indicate that when the flagellar motor is not able to propel the flagella or when the bacteria do not have a fully functional flagellum, pathogenic *Leptospira* sp. are impaired in their ability to move, which as a consequence impairs the dissemination in host tissues and prevents disease progression.

### LPS As a Virulence Factor

Several mutant strains in genes annotated as LPS biosynthesis genes showed a NV phenotype (Table 2; Figure 2), demonstrating that this cell wall component is crucial for virulence. The LPS is a complex molecule encoded by a huge locus larger than 100 kb, rather conserved among pathogenic *Leptospira* sp. strains (106). LPS is constituted of three parts, the lipid A moiety that anchors the LPS in the OM, a conserved core oligosaccharide, and a polysaccharide component, the immunogenic O antigen, which is highly variable in composition and length. Antigenic diversity constitutes the basis of the serological classification of pathogenic *Leptospira* sp. Two mutants in the LPS biosynthesis locus of the *L. interrogans* Manilae strain L495, M895 altered in *Lman-1456* (LA1641) and M1352 altered in *Lman-1408* (94) have been obtained by random mutagenesis (94). Both of the genes were of unknown function and *Lman-1408* was specific to the Manilae strain. According to electrophoretic profiles after silver staining, M895 was truncated in the O antigen but not M1352. Both presented altered recognition by a polyclonal serum against the WT Manilae L495 strain and were NV in the hamster model, even at the very high dose of 10^7^ bacteria. Interestingly, it was later shown that both mutants M895 and M1352 were also impaired in their ability to colonize the kidney of BALB/c mice (107) (Table 3).

Two other mutant strains of *L. interrogans* serovar Manilae L495 have been obtained by random mutagenesis in two different genes, both annotated *lpx*D, coding for LpxD, the *N*-acyltransferase of lipid A biosynthesis (Table 3). In different bacteria, change in temperature upon entry in the host regulates the function of LpxD, which modifies the number or length of acylated chains in the lipid A. Since LPS is an abundant component of the OM, the modified hydrophobicity of the lipid A can directly influence the OM fluidity. This mechanism appears to be important for the host adaptation to temperature changes, and therefore to virulence (108). The mutant strain in the gene la0512 (90) annotated *lpx*D1, was NV in gerbils. Compared to the parental strain or to the second mutant lpxD2, still V, the LpxD1 mutant had altered growth at 37°C, although growth at 30°C was not altered. The LpxD1 mutant also had reduced resistance to the antimicrobial peptide polymyxin B at 37°C, reflecting altered OM integrity. Complementation of the LpxD1 mutant restored all altered phenotypes, as well as virulence (90). However, the structure of the lipid A from the WT and mutant strains grown *in vitro* at different temperatures were analyzed by Maldi-MS but surprisingly it did not show major modifications of the structure (90), found identical to the lipid A structures from *L. interrogans* serovar Icterohaemorraghiae strain Verdun and serovar Pomona strain L170 (79). These results raised questions about the biochemical function of the *Leptospira* sp. enzymes annotated as LpxD that potentially could acylate components other than lipid A. Nevertheless, the LPS of the LpxD1 mutant was not recognized by a polyclonal serum against L495, suggesting that the enzyme is indeed modifying the LPS structure. Moreover, in *L. interrogans* serovar Copenhageni strain L1-130, able to cause acute disease in the guinea pig and asymptomatic chronic renal colonization in rats, it has been shown that the O antigen content of the LPS is decreased in bacteria retrieved from the liver of moribund guinea pigs, compared to the LPS of bacteria retrieved from the kidneys of rats. The latter also showed the same electrophoretic profiles than the LPS prepared from bacteria grown in EMJH (51). These data suggest that the modulated expression of the O antigen part of the LPS is important for virulence in the acute model of infection. These studies showed that *Leptospira* sp. LPS is crucial for virulence although the underlying mechanism remains to be understood. Potentially, the decreased O antigen could help the bacteria escape early antibody response, shown in mice to occur as soon as 3 days post-infection (44). At the chronic phase in the rat, the O antigen is fully expressed, possibly protecting *Leptospira* sp. from the IgG response. We could speculate that this complete form of LPS could provide bacteria shed in urine with some advantage in survival in the environment or in the early phase of infection.

### LoA22, Mammalian Cell Entry (Mce), and ColA OMPs Are Virulence Factors

The OMP LoA22, whose function is still unknown, is one of the few OM candidate protein (Table 3; Figure 2) shown to be a virulence factor (87). LoA22 was found to be the most upregulated OMP in the liver of moribund guinea pigs infected with *L. interrogans* serovar Copenhageni (109). In hamster, the *loA22* mutant of the *L. interrogans* serovar Lai was NV, and the complementation restored the virulence. Interestingly, the same mutant was found only attenuated in guinea pigs, since it did not kill the animals and was not found in the liver but still colonized the kidneys (87). These results suggest that LoA22 displays an important role at the acute phase of the infection during multiplication in blood or dissemination in tissues, but may not be a key player in permeation of the tubules.

Other OMPs that might be considered as involved in invasion of *Leptospira* sp. are the Mce protein (98), which has homologs in other pathogenic bacteria, and the collagenase A protein ColA (97), encoding for a protein involved also in host–pathogen interactions during invasion and transmission. Both mutants for Mce and ColA proteins are attenuated.

### LipL32, LipL41, and LigB OMPs Are Not Virulence Factors

All the other OM components tested were not involved in virulence (Table 4; Figure 2). Particularly striking is the case of LipL32, which is the most abundant lipoprotein of the OM and is highly immunogenic, and also expressed during acute infection of guinea pig (109), but whose expression is downregulated in the blood of OF1 mice and hamsters (110). Knockdown of LipL32 did not abrogate virulence of the strains tested in the acute and the chronic model of the disease, leading to death of the hamsters and kidney colonization of rats in the same extent of the WT strain (95). Moreover, this mutant was still V when administered through the conjunctiva, showing that Lip32 is not necessary in the first steps of the infectious process of penetration through the mucosa (95). It is important to note that absence of LipL32 has a deep impact on the cell, with 46 genes modulated, as shown by microarray analysis (95), although LipL32 does not affect the virulence process. Likewise, the third most abundant OM lipoprotein LipL41 is not necessary for virulence of *Leptospira* sp. in the acute model of the disease, since all the animals infected with the mutant strain succumbed to the infection (96). These results, both from LipL41 and LipL32, suggest either that these lipoproteins, of unknown function, may be important for the survival of *Leptospira* sp. in the environment or that the numerous lipoproteins encoded in the *Leptospira* sp. genome could have redundant functions, which could allow bacteria mutated in a single lipoprotein gene to retain virulence. Notably, a targeted mutant of *L. interrogans* serovar Copenhageni strain Fiocruz L1-130 in the protein LigB, considered to be involved in *Leptospira* sp. adhesion to the host and upregulated 24 h post-infection *in vivo* in blood of both OF1 mice and hamsters (110), retained its virulence in both models of acute and chronic disease (99) (Table 4).

### Other Virulence Factors

The catalase KatE (104) is another factor linked with loss of virulence, which could be involved in escape of bactericidal activity from neutrophils and macrophages due to detoxification of ROS produced by phagocytes. This hypothesis is supported by the fact that KatE is located in the periplasmic space of *Leptospira* sp. cell wall where it can participate in ROS resistance. Mutants of the chaperone HtpG (102), which is related to virulence in other bacterial species and of the protein LruA, a 28 kDa surface-exposed lipoprotein that might interact with the serum apolipoprotein A1 (103) lead to an attenuated phenotype in the hamster model of acute infection.

Also, a putative regulatory locus of *L. interrogans* Manilae L495 (*lb139*), important for multiple gene regulation, including motility genes has been recently shown to be a virulence factor. Indeed, the lb139 insertion mutant did not kill hamsters, nor did it colonize the organs (101).

A high-throughput method has been developed to screen for new virulence factors in *L. interrogans* serovar Manilae strain L495. The technique consists in injecting in the hamster or in the BALB/c model of renal colonization, a pool of mutants obtained by random mutagenesis. Then, presence of each mutant is tested by specific PCR, in the pool of *Leptospira* sp. retrieved in culture from the kidneys of moribund animals, compared to the pool of mutants grown in culture. The mutants not found in organs are assumed to be NV (107). Recently, an improved version of this technique, allowing for direct quantification of mutants in organs and blood, and therefore able to assess their relative fitness, has been performed in hamsters. A new virulence factor, an adenylate/guanylate cyclase gene, has been identified. This mutant was attenuated when tested individually in hamsters (100). Interestingly, a soluble adenylate cyclase was previously identified as a putative virulence factor using comparative sequencing of a V strain of *L. interrogans* serovar Lai strain 56601 versus an isogenic culture derivative strain. This protein was shown *in vivo* to be highly upregulated in the hamster model compared to the EMJH culture, and *in vitro* to elevate the intracellular cyclic AMP in macrophages (111), therefore potentially reducing the host innate TNF response, as previously shown in *Mycobacteria*-infected macrophages (112).

Another recent study using RNA-Seq compared the transcriptome of V *L. interrogans* serovar Copenhageni grown *in vitro* in EMJH, to *Leptospira* sp. cultivated *in vivo* within a dialysis chamber implanted in the peritoneal cavity (DMC) of Sprague-Dawley rats (113), mimicking the host adapted state. 10 days post inoculation, motile *Leptospira* sp. harvested in exponential growth were used to prepare RNAs. Other than a core of genes identically regulated between the two conditions, the authors found 166 genes differentially expressed, most of them being specific of the pathogenic but not the saprophytic strain (113). The analysis of the upregulated genes, including a comparison with other serovars, provides a comprehensive picture of the genes important in the host–pathogen interactions. Some genes already known as virulence factors, such as collagenase A, were expressed 50 times more in DMC than *in vitro*. Also found in these studies were lipoproteins, hemolysins, and flagellar components, but most of them are unknown genes that remain to be studied (113).

A summary of the virulence factors of leptospirosis is presented in Figure 2.

## Conclusion and Future Studies

Animal models, such as guinea pigs, gerbils, and in particular hamsters, have been instrumental to understand the pathophysiology of lethal leptospirosis, as well as to get insight into the *Leptospira* sp. genes involved in virulence. Using these animal models, high-throughput methods, relatively easy to perform, spare animal use and can be applied each time a new random mutagenesis (99, 114, 115) is performed and might facilitate the discovery of new virulence factors of pathogenic *Leptospira* sp. These models have also been very important to test vaccines, which was not the focus of the present review. Vaccines for leptospirosis have been recently reviewed by Adler (116).

The overlooked mouse model has recently proved its usefulness to provide insight both in sublethal and chronic leptospirosis, in particular the host immune mechanisms that control pathogenic *Leptospira* sp., thus unlocking an avenue of research into the immunological mechanisms of susceptibility or resistance to *Leptospira* sp. infection. Moreover, these versatile murine models are appropriate to test new vaccine candidates and drugs to treat leptospirosis. In the near future, the availability of CrispR/Cas 9 technology should provide new tools to design both new deficient mice and targeted mutants of *Leptospira* sp. to study the contribution of unique genes in host–pathogen interaction studies. The new genetic tools available should also help shift research from the current descriptive forms to a more complex mechanistic evaluation of the pathophysiology induced by pathogenic *Leptospira* sp. with the goal of finding the function of specific genes. Indeed, transgenic mice models, devoid of certain genes or cell subsets that can further be studied in different compartments using the Cre/Lox system, are unique means to understand the crucial question of how *Leptospira* sp. overcome their hosts’ immune responses.

Alternative, non-traditional models could also contribute to the understanding of chronic infection. Natural outbred strains of mice (*Mus musculus*, Swiss Webster) can be used to study how pathogenic *Leptospira* sp. establish effective infection in reservoir hosts without causing disease. Natural hosts of human pathogens such as *Leptospira* sp. develop tolerance to the pathogen and are usually asymptomatic when infected, which is the opposite to an immune response developed by the “accidental” host (human) that succumbs to the pathogenic effect of the agent. These non-traditional animal models could be used in comparative studies with strains of mice engineered to be susceptible to *Leptospira* sp. dissemination. In the “One Health” context, research on resistance and tolerance in reservoir hosts is required for development of Public Health measures targeting natural reservoirs and for understanding the mechanisms of human leptospirosis and other infections.

Human leptospirosis studies are mostly epidemiologic, using blood or urine as biological samples, and can only provide a limited amount of information about mechanisms of host–pathogen interaction. Long-awaited human polymorphisms studies have not provided clear answers about innate immune genes involved in protection against leptospirosis. Therefore, notwithstanding ethical concerns about experimentation in humans and animals, increased regulations, and justified restrictions about animal use in research, it is extremely important to keep developing and using animal models of leptospirosis to further our understanding of the disease as well as to identify vaccine candidates, therapeutics, and diagnostic assays. New vaccines and immuno- or other therapies to prevent rapid progression of *Leptospira* sp. through tissues and colonization of the kidney could help prevent severe cases of the disease and save lives. We may think that the clinical picture of leptospirosis is rather well known, but some unexpected findings may challenge our current knowledge. For example, *Leptospira* sp. were found in lungs of asymptomatic wild rats carrying the spirochete in their kidneys, adhering to the ciliated surface of the bronchi (117). In addition to the intraperitoneal route of infection, necessary to precisely determine infectious dose, more physiological routes of infection, such as the conjunctival, transdermal, and other mucosal routes that have recently been studied in rats (60) and hamsters (27), should be investigated in different hosts, including mice, gerbils, and guinea pigs. Using a natural route of infection may be how we could develop a long-awaited model for neuroleptospirosis (118). In addition, disease progression might be different if *Leptospira* sp. is deposited in the peritoneum or if it is deposited in skin or mucosal surfaces and expected to waddle through the natural barriers including skin, extracellular matrix, and the immune system before it reaches target organs.

As an alternative to mammal use, the development of the *Zebrafish* model of leptospirosis (77, 83) should in the future also help with innate immunity studies. Indeed, the NF-κB and Interferon signaling pathways are conserved through evolution, and the generation of genetic mutants in Zebrafish is amenable (119). Likewise, the development of a *Caenorhabditis elegans* model, that is well established for characterization of molecular mechanisms involved in pathogen activation of innate immune pathways, as well as pathological mechanisms, would be welcomed to study leptospirosis since the genome of this worm is reduced and the genetic tools are available (120). However, it is not known yet whether *Leptospira* sp. are pathogenic for this nematode, like are other human or zoonotic pathogens such as *Coxiella, Salmonella*, or *Pseudomonas* sp.

Leptospirosis is a zoonosis, and humans are just one among many other vertebrates to be affected by the disease (3). Epidemiologic data show that less than 7% of people infected with pathogenic *Leptospira* sp. die from severe forms of leptospirosis (3, 121). The current view that humans mostly suffer from acute severe disease is outdated and has recently been questioned given that clinical manifestations of human disease recapitulate all the different clinical profiles and stages found in animals. It ranges from asymptomatic to lethal, encompassing sublethal as well as chronic forms. Indeed, chronic renal infection that occurs in endemic areas of leptospirosis and asymptomatic disease, revealed by seropositive serum against *Leptospira* sp., has recently been shown to favor chronic kidney disease (3, 9, 59). Interestingly, all these clinical forms can be studied using the animal models presented in this review. These data suggest that the dichotomy between acute and chronic diseases may be a man-made artifact and that the classification of leptospirosis according to the symptoms may not represent the continuum of a biphasic disease, with blood dissemination and seeding of target organs with *Leptospira* sp., which may be severe enough to impair organ function and cause death, or depending on the capability of the host immune response to control *Leptospira* sp. dissemination, the disease can be mild allowing for natural recovery and kidney colonization.

Leptospirosis is a neglected disease with a limited number of groups working on it. Compared to *B. burgdorferi*, another spirochete that causes Lyme disease, we still lack a lot of useful tools, such as GFP or RFP fluorescent *Leptospira*, sp. allowing for efficient imaging in cells and tracking of bacteria in the host. Hopefully, recombinant labeled strains will be constructed, potentially using the replicative plasmid pMaori, adapted to pathogenic *Leptospira* sp. (105), or CrispR/Cas 9 technology. Although not allowing sensitive imaging in organs, bioluminescent strains have been developed and may also give some clues about the kinetics and localization of *Leptospira* sp. in different hosts, as well as horizontal or vertical routes of transmission of *Leptospira* sp. within hosts, and in the environment (38).

An important topic that until now has been overlooked is the species-specificity adaptation of *Leptospira* sp. to their hosts. We know that some *Leptospira* sp. serovars are more commonly associated with particular hosts, such as Ballum with mice, Canicola with dogs, Hardjo with cattle, or Icterohaemorrhagiae with rats (122, 123). Lately, sequencing of hundreds of leptospiral genomes highlighted specific features of pathogenic strains (123, 124), but the basis of the host preferences of *Leptospira* sp. is unknown. Immune factors, such as TLR4 and TLR2, contribute to the resistance of the host, and most probably shape the nature of the pathogen interaction with a particular species. On the other hand, recent work showed that the LPS of pathogenic and intermediate *Leptospira* sp. were different (125). Therefore, future structure–function and expression studies of TLRs and NLRs from different animals toward molecules from different serovars should give interesting clues about the sensitivity of a given species. Hopefully, it would explain the leptospirosis symptoms, and target organs that may vary from one host to another, such as the ocular manifestation in horses, placenta infection in cattle, or renal insufficiency in dogs, for example.

### Summary

In addition to other animal models, congenic and transgenic mouse models of leptospirosis offer a myriad of possibilities to advance knowledge of host–pathogen interactions especially as it concerns the study of early innate and adaptive immune responses to pathogenic *Leptospira* sp. Mice can be used for profiling immune responses to dose-dependent sublethal, lethal, and chronic leptospirosis, which could inform understanding of the natural disease progression in longitudinal studies. From an applied research standpoint, mice are well-accepted experimental models that can be readily adapted to higher throughput testing of either vaccine candidates or therapeutic options.

## Author Contributions

MGS wrote the introduction and summary, contributed to the writing of the physiology part, and edited the manuscript and tables. IS contributed to the writing of the virulence part and did the figures and tables. CW wrote and edited the manuscript and tables.

## Conflict of Interest Statement

The authors declare that the research was conducted in the absence of any commercial or financial relationships that could be construed as a potential conflict of interest.

## Abbreviations

sp., species; ko, knockout; TLR, toll-like receptor; DAF-1, decay-accelerating factor 1; LPS, lipopolysaccharide; NLR, nod-like receptor; PBMC, peripheral blood mononuclear cell; BMM, bone marrow derived macrophages; Ig, immunoglobulin; LRR, leucine rich repeat; Na/K-ATPase, potassium pump; GLP, glycolipoprotein; IL, interleukin; NO, nitric oxide.
